# Ripple density resolution dependence on ripple width

**DOI:** 10.1371/journal.pone.0270296

**Published:** 2022-07-22

**Authors:** Alexander Ya. Supin, Olga N. Milekhina, Dmitry I. Nechaev, Marina S. Tomozova

**Affiliations:** Institute of Ecology and Evolution, Russian Academy of Sciences, Moscow, Russia; All India Institute of Speech and Hearing, INDIA

## Abstract

The goal of the study was to investigate how variations in ripple width influence the ripple density resolution. The influence of the ripple width was investigated with two experimental paradigms: (i) discrimination between a rippled test signal and a rippled reference signal with opposite ripple phases and (ii) discrimination between a rippled test signal and a flat reference signal. The ripple density resolution depended on the ripple width: the narrower the width, the higher the resolution. For distinguishing between two rippled signals, the resolution varied from 15.1 ripples/oct at a ripple width of 9% of the ripple frequency spacing to 8.1 ripples/oct at 64%. For distinguishing between a rippled test signal and a non-rippled reference signal, the resolution varied from 85 ripples/oct at a ripple width of 9% to 9.3 ripples/oct at a ripple width of 64%. For distinguishing between two rippled signals, the result can be explained by the increased ripple depth in the excitation pattern due to the widening of the inter-ripple gaps. For distinguishing between a rippled test signal and a non-rippled reference signal, the result can be explained by the increased ratio between the autocorrelated and uncorrelated components of the input signal.

## Introduction

Stimuli with rippled spectra have been successfully used to test the spectro-temporal resolution of hearing. However, the estimates of the resolution depend on the parameters of the rippled signals used. One important parameter is the ripple form.

A simple ripple form is a harmonic form, in which the spectral ripples are defined by a sine or cosine function of either the frequency (linearly spaced ripples, with the density specified by the number of ripples per linear frequency unit, e.g., ripples/kHz) or the frequency logarithm (logarithmically spaced ripples, with the density specified by the number of ripples per log frequency units, e.g., ripples/oct).

In addition to harmonic ripples, a variety of other ripple forms have been investigated. In earlier studies, rippled stimuli were generated by adding white noise to delayed copies [1–3]. This procedure produces noise with the rippled spectrum, with a ripple frequency spacing of 1/*δτ*, where *δτ* is the delay. The power spectrum of this stimulus is a raised cosine function of the frequency (linear ripple frequency spacing), i.e., the amplitude spectrum is the square root of the raised cosine function. Stimuli with linearly spaced ripples produce a repetition pitch, which corresponds to the pitch of a tone frequency equal to *δf*. The repetition pitch appeared at ripple densities less than 20 ripples/kHz [2]. This limit can be taken as an estimate of the ripple-density resolution for the particular ripple pattern. With this stimulus-generation technique, ripple forms were varied by the number of “delay and add” iterations, yielding a stimulus known as iterated rippled noise: the more iterations there are, the narrower the ripples. The narrower the ripples (as a result of more iterations), the stronger the repetition pitch [4, 5].

In later studies, rippled stimuli were used to evaluate the spectral or spectro-temporal resolution of hearing, regardless of the repetition pitch. In general, stimuli with logarithmically spaced ripples (ripple density specified in ripples/oct) were used [6–12], since this ripple pattern better fits the distribution of cochlear frequency tuning along the frequency axis. The resolution of the ripple density depended on the discrimination task. When the resolution was assessed by distinguishing two rippled stimuli with equal ripple densities but different ripple phases, the resolution was less than 10 ripples/oct [6, 7]. When the resolution was assessed by distinguishing between a rippled stimulus and a non-rippled stimulus, it was several times higher: nearly 60 ripples/oct [11], 26 ripples/oct [13], or more than 34 ripples/oct [14]. However, the influence of the ripple width on the ripple density resolution was not investigated in these studies.

The goal of the present study was to investigate how various ripple widths influenced the ripple density resolution. The influence of the ripple width was investigated with two experimental paradigms: (i) distinguishing between rippled test signals and rippled reference signals with different patterns of ripple phases and (ii) distinguishing between a rippled test signal and a non-rippled reference signal.

## Methods

### Listeners and experimental conditions

For the present study, seven normal-hearing listeners (four males and three females) were available. The listeners ranged in age from 23 to 39 years. No participants had any history of hearing disease, and all had hearing thresholds of less than 10 dB in the range of 1 to 4 kHz, as measured by an automatic audiometer AA-02 (Biomedilen, St. Petersburg, Russia). During the measurements, the listener sat comfortably in a sound-proof cabin (MINI 350, IAC, Germany) that attenuated external sounds by at least 40 dB.

The experiments were approved by the Ethics Committee of the Institute of Ecology and Evolution, where the study was performed. A permit was given for sound exposures of up to 70 dB SPL, with an everyday sound exposure level of 100 dB re 1 μPa^2^s. All the participants signed informed consent forms before participating in the study under the conditions specified above.

### Signal parameters

Three types of signals were used for measurements: the test and two reference signals.

**Fig 1:**
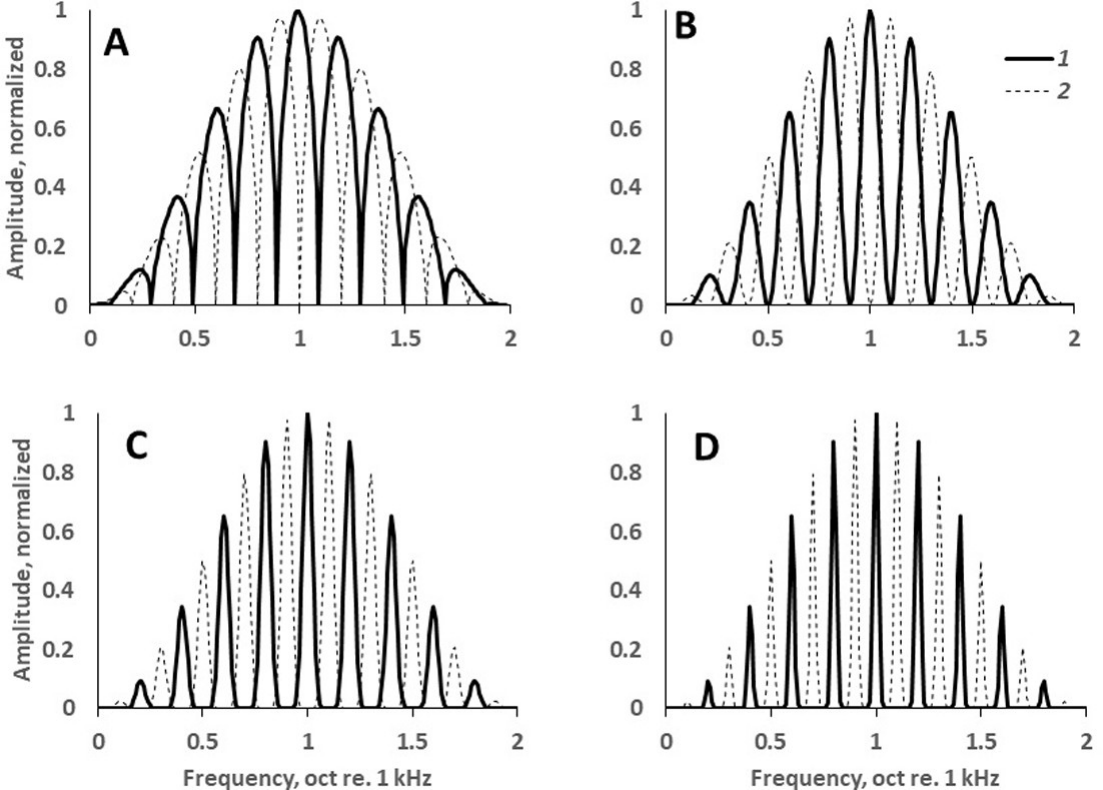
Examples of spectra with various ripple widths. The spectra are obtained at various values of the exponent exp in Eq (1). All the spectra are centered at 2 kHz and have a ripple density of 5 ripples/oct. *1* and *2* –two versions of the spectra, where the ripples have opposite phases. *Exp* values of 0.25 (A), 1 (B), 4 (C), and 16 (D) result in ripple widths of 64, 37, 20, and 13% of the ripple spacing, respectively.

**Fig 2:**
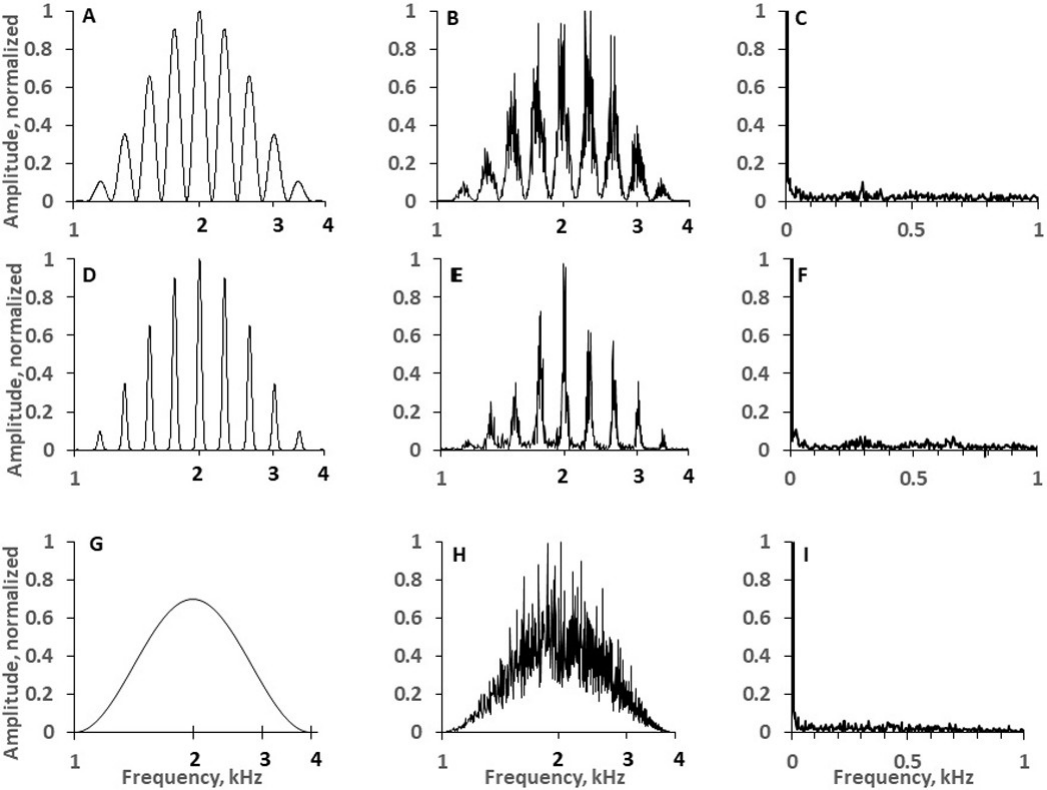
Filter waveforms and spectra of the synthesized signals. A: Filter form for a signal of a ripple density of 5 ripples/oct and ripple width of 37%. B: The spectrum of a 400-ms sound burst synthetized using this filter. C: The spectrum of the burst envelope. D-F: The same as A-C for a rippled signal of 5 ripples/oct, 16%. ripple width. G-I: The same for a flat signal.

The *test signals* (Figs 1 and 2A, 2B, 2D and 2E) had band-limited spectra with logarithmically spaced ripples with various ripple densities and widths. The spectra had an envelope that was one two-octave wide cycle of a raised cosine function of a frequency logarithm. The cycle was centered at a frequency of 2 kHz and extended from 1 to 4 kHz.

Within the envelope, the spectrum had ripples that were defined as the varied power of a raised cosine function:

$$A(f)={\left[0.5+0.5\;\text{cos}\;(2\pi D\;{\text{log}}_{2}\;f)\right]}^{exp},$$ (1)

where *A*(*f*) is the normalized spectral amplitude (varied from 0 to 1), *D* is the ripple density (ripples/oct), *f* is the frequency (kHz), and *exp* is the exponent index. The constant 0.5 and the factor 0.5 serve to make the cosine function raised to a range from 0 to 1. The ripple density *D* was varied stepwise: 2, 3, 5, 7, 10, 15, 20, 30, 50, 70, 100, 150, and 200 ripples/oct (a quasi-logarithmic scale with 6 steps per log_10_ unit). The exponent *exp* was also varied stepwise: 0.25, 0.5, 1, 2, 4, 8, 16, and 32. The obtained ripple widths were characterized by their equivalent rectangular bandwidths (ERBs) defined as a width of a rectangular spectrum band that contains the same power as the ripple of the same peak power:

$$ERB=\frac{1}{P_{peak}}\int_{-lt}^{ht}P(f)df,$$ (2)

where *f* is frequency, *P*(*f*) is the spectrum power at a frequency *f*, *P*_*peak*_*−*peak power of the ripple, *lt* and *ht*–frequencies of the lower and higher ripple troughs, respectively. ERBs were specified as percentage of the ripple frequency spacing. For the exponents specified above (from 0.5 to 32), the ripple ERBs were 64, 50, 37, 28, 20, 16, 13, and 9% of the ripple frequency spacing, respectively. In particular, at *exp* = 1 (harmonic ripples), ERB = 37%; at *exp* = 0.5 (ripples resulting from a single “delay and add” procedure), ERB = 50%.

The ripple phase was inverted every 400 ms, i.e., the positions of the ripple peaks and troughs on the frequency scale were reversed (spectrum versions *1* and *2* in Fig 1). Each signal included six segments with alternating ripple phases, resulting in overall duration of 2400 ms.

*The rippled reference signal* had the same frequency band (with respect to both the centroid and bandwidth), duration, level, and ripple density as the test signal (Fig 2A, 2B, 2D and 2E).

It differed from the test signal by a constant ripple phase that was not changed throughout the signal duration.

*The flat reference signal* had the same frequency band (with respect to both the centroid and bandwidth), duration, and level as the test signal, but it had no ripples in its spectrum (Fig 2G and 2H).

### Signal generation and monitoring

All the signals were digitally synthesized at a sampling rate of 32 kHz, with a total of 2^17^ = 131072 samples (4096 samples/kHz). The signals were synthesized on a standard personal computer using a custom written program (virtual instrument) designed using LabVIEW software (National Instruments, Austin, TX, USA).

To obtain a desirable signal, a Gaussian digital sequence was transferred through a digital filter. The filter determined both the envelope and (when applicable) the ripple pattern of the signal spectrum.

To generate a test signal, two filters with opposite ripple phases were used. Every 400 ms, the Gaussian sequence was switched from one filter input to the other; the filter outputs were summed. It should be noted that the switch was performed at the filter inputs, not the outputs. Therefore, at the phase reversals, the spectrum of the output signal never extended beyond the spectrum band determined by the filters. The signal included six segments produced by the filters with opposite ripple phases.

To generate a reference signal, one filter was used, with either a rippled or non-rippled frequency response.

The digital generation included the following steps. The impulse response of the filter was obtained by inverse Fourier transform of its shape. White noise was generated as a Gaussian digital sequence. The white noise was passed through a finite impulse response digital filter. The filtering was performed by convolution of the Gaussian digital sequence with the filter impulse response.

The spectra of the generated signals differed from the frequency responses of the filters used (Fig 2A, 2D and 2G) due to the presence of fluctuations caused by random fluctuations in the Gaussian noise. Despite these fluctuations, both the spectrum envelopes and the ripple patterns defined by the filters were reproduced in the generated signals (Fig 2B, 2E and 2H). Because of the non-uniform (logarithmic) ripple spacing in the signals, their envelopes did not feature obvious periodicity: at frequencies corresponding to the ripple frequency spacings, amplitudes of spectral components of the envelopes were of the same order of magnitude as random fluctuations outside this frequency band (Fig 2C, 2F and 2I).

The digitally synthesized signals were digital-to-analog (D/A) converted by a 16-bit converter on an NI-DAQ-6215 data acquisition board (National Instruments). The analog signals were played diotically (equally to both ears) at a level of 70 dB SPL through Schenheiser HD580 (Sennheiser, Wedemark, Germany) headphones. The parameters of the signals were monitored and calibrated using a RA0039 (G.R.A.S., Holte, Denmark) ear simulator.

Level roving was not used because the listeners found it difficult to detect a difference between the test signal and the two reference signals when all three signals had different levels. The signal levels and waveforms were monitored at the output of the headphones.

### Procedure

The ripple density resolution was measured using a three-alternative forced-choice paradigm. In each trial, the listener heard three successive sound signals, which were each 2400 ms long and separated by 100-ms pauses: one test signal and two reference signals. The order of the signals during the trial (whether the test signal was in the first, second, or third position) varied randomly for each trial. The task of the listener was to determine which of the three signals differed from the other two, i.e., which was the test signal. The listener was not instructed to pay attention to any particular quality of the signals; thus, he or she could rely on any cue to distinguish the signals.

In each trial, the starting ripple density was 2 ripples/oct; this ripple pattern was resolved by all the listeners at any ripple width. Then the ripple density was adaptively varied trial-by-trial using a “two-up, one-down” version. After two successive hits (correct detection of the test signal), the ripple density in the next trial was increased by one step; after each mistake, the ripple density in the next trial was decreased by one step. This adaptive variation tracked the ripple density around a value that results in a probability of correct detections equal to 0.5^0.5^ = 0.71 [15]. This probability was taken as the ripple density resolution as close to the midpoint of 0.67 between an unerring test detection of 1.0 and 0.33, the probability of a random guess when the test signal was undetectable. The tracking continued until 10 reversal points (transition from the ripple density increasing to decreasing and back) were obtained. The geometric mean of these 10 points was taken as an estimate of the resolution for that run.

The ripple width was kept constant during each measurement run and was varied randomly between runs by the steps specified above.

### Data processing and statistics

For each of the listeners, the measurements of the ripple density resolution for each ripple width were repeated three times. The arithmetic mean of these three results was taken as an estimate of the resolution for that listener. The final result for a particular ripple width was taken as the interindividual mean and standard deviation.

Statistical processing was performed using Prism (GraphPad, San Diego, CA) software.

## Results

The main results of varying the type of reference signals and ripple width were: (i) higher ripple density resolution for flat than for rippled reference signals and (ii) dependence of the ripple density resolution on the ripple width: the narrower the width was, the higher the resolution.

Both Shapiro-Wilk and Kolmogorov-Smirnov tests showed that inter-individual distributions of ripple density resolutions were normal for both rippled and flat reference signals.

Higher resolution for distinguishing between a rippled and a flat signal than between two rippled signals was statistically significant with a large effect size, as revealed both by ANOVA (*F* = 24.6, *df* = 1, *Η* = 0.73, *P* = 0.002) and by Friedman non-parametric test (p < 0.001).

**Fig 3:**
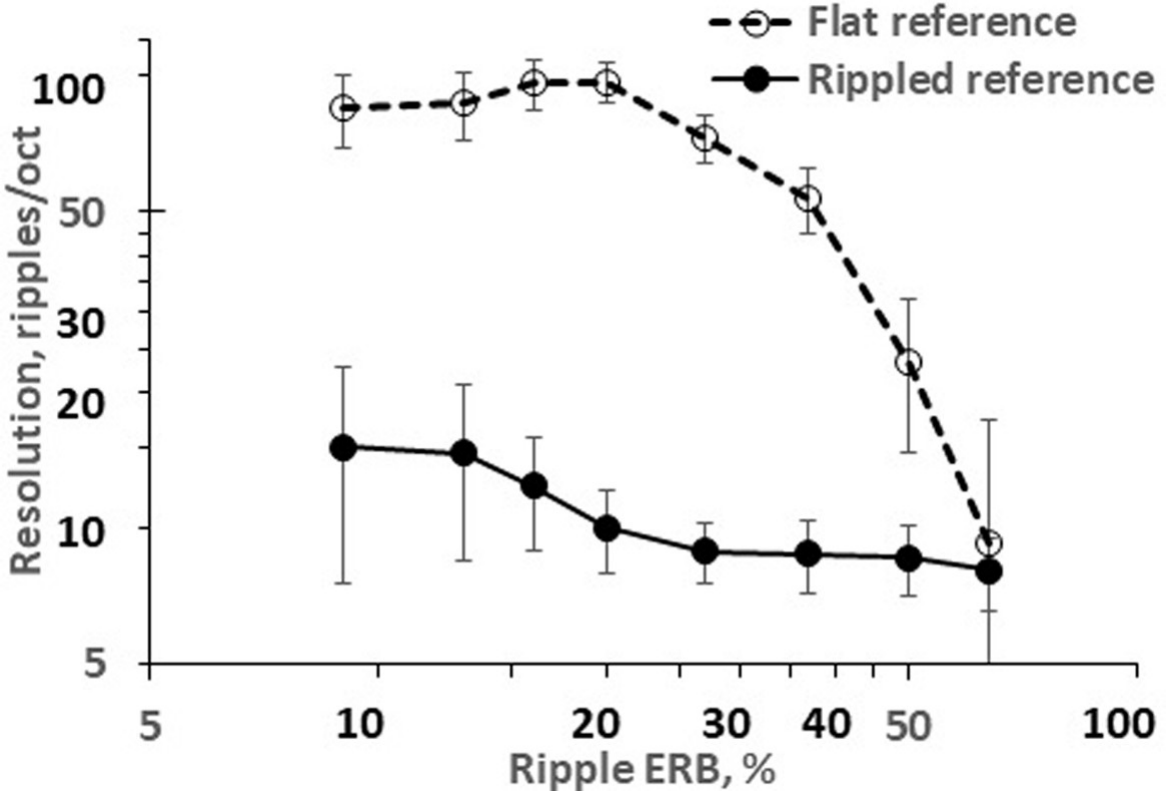
Ripple density resolution as a function of the ripple width. Inter-individual means; data for rippled and flat reference signals are shown, as indicated in the legend. The error bars represent interindividual standard deviations.

The dependences on the ripple width were observed both for distinguishing between two rippled signals and for distinguishing between a rippled test signal and a flat reference signal (Fig 3). For distinguishing between two rippled signals, increasing the ripple width from 9 to 64% reduced the ripple density resolution (inter-individual means and standard deviations) from 15.1 ± 7.6 to 8.1 ± 1.5 ripples/oct. The dependence on ripple width was statistically significant with a large effect size as revealed both by ANOVA (*F* = 8.41, *df* = 7, *Η* = 0.68, *P* < 0.001) and by Friedman non-parametric test (*p* < 0.001). For distinguishing between a rippled signal and a flat signal, the highest resolutions of 96.8 ± 9.6 ripples/oct was observed when the ripple width was 20%; for the narrowest ripples of 9%, the resolution was 85.0 ± 15.3 ripples/oct. Increasing the ripple width to 64% decreased the ripple density resolution to 9.3 ± 8.0 ripples/oct. The dependence on ripple width was statistically significant with a large effect size by ANOVA (*F* = 75.9, *df* = 7, *Η* = 0.86, *P* < 0.001).

The rate of dependence was demonstrated by the regression analysis Since inter-individual distributions of ripple density resolutions were normal (see above), none of the listeners were considered outliers, and results for all seven listeners were taken for the regression analysis. For rippled reference signals and within a ripple width range from 13% (a breakpoint of the resolution-vs.-width function) to 64%, the regression slope and its standard error (SE) were –0.10 ± 0.02 (ripples/oct)/% (P = 0.03). For flat reference signals and within a width range from 20% (a breakpoint) to 64%, the regression slope and SE were –2.04 ± 0.17 (ripples/oct)/% (P < 0.001).

## Discussion

It has been argued [11, 13, 14] that for different discrimination tasks, different mechanisms dominate. For distinguishing between two rippled signals that have different ripple positions on the frequency scale, spectral cues are more effective. For distinguishing between a rippled signal and a non-rippled signal, temporal cues provide higher resolution. Because of the differences between these two mechanisms, the effects of the ripple width differ when discriminating between two rippled signals or between rippled and non-rippled signals.

The role of envelope modulation of rippled signals has been shown for broadband stimuli with linearly spaced ripples, for ripple spacing below 33 Hz [16]. Stimuli used in the present study (narrowband, with logarithmically spaced ripples) feature negligible envelope (See Fig 2C and 2F). So, below the effects of ripple width on the spectral and temporal mechanism are considered.

### Spectral cues

Ripple density resolution models based on spectral cues assume that passing a rippled signal through a bank of frequency-tuned filters results in an excitation pattern which is a distribution of excitation across the frequency representation in the auditory system. An excitation-pattern model was suggested for distinguishing tone signals [17]; however, this model can also be applied for rippled-spectrum signals. For rippled-spectrum signals, the excitation pattern is also rippled; however, its ripple depth is lower than that of the signal because of the integration of the spectral components in the filter passbands. The higher the ratio of the ripple density to the filter quality, the lower the ripple depth. The rippled spectrum pattern can be resolved when the ripple depth exceeds a certain threshold.

**Fig 4:**
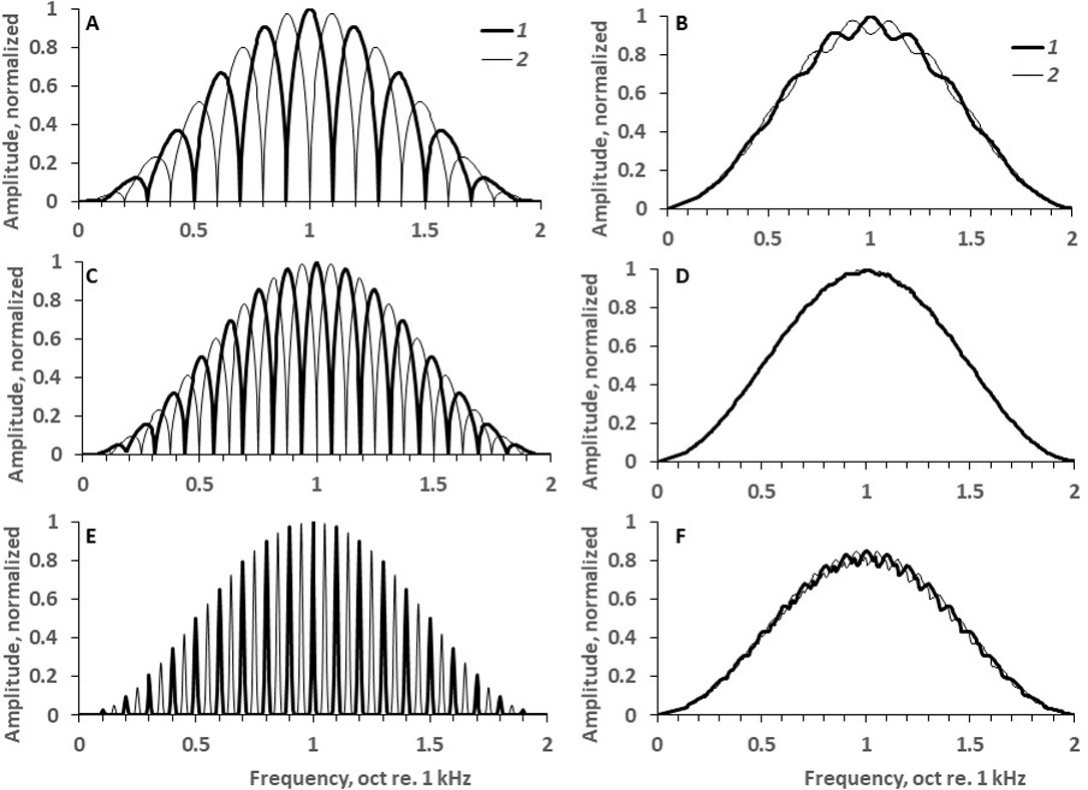
Simulation of excitation patterns. A: input signal spectrum of 5 ripples/oct and a ripple width of 64%; *1* and *2* –spectra with opposite ripple phases. B: Excitation pattern simulated for a filter passband of 0.17 oct. C and D: The same for 10 ripples/oct and a ripple width of 64%. E and F: The same for 10 ripples/oct and a ripple width of 9%.

Fig 4 illustrates several rippled spectra and corresponding simulated excitation patterns appearing due to transferring the spectra through a bank of filters. The filter form was taken as a *roex* function [18]. For a frequency of 2 kHz, the formula developed by [19] predicts a filter passband of 237 Hz, which corresponds to 0.17 oct. The simulated excitation pattern (Fig 4B) obtained by convolution of the spectra (A) with the filter form specified above has a ripple depth of 0.8 dB. Experimental results showed that this combination of the ripple density and width was resolvable in a task to discriminate between two rippled signals because the found threshold was 8.1 ripples/oct (see Fig 3, the result for 64% ripple width). Increasing the ripple density to 10 ripples/oct while maintaining the same ripple width of 64% (Fig 4C) resulted in an excitation pattern with a ripple depth of 0.15 dB (Fig 4D); this input spectrum was not resolvable for the same discrimination task because the density of 10 ripples/oct was beyond the threshold (see Fig 3); so, the model predicted that a ripple depth of 0.15 dB was unresolvable. Narrowing the ripple width to 9% increased the inter-ripple gaps and restored the ripple depth in the excitation pattern to 0.7 dB. This ripple pattern was resolvable (see Fig 3, the result for 9% ripple width). Based on these examples, we hypothesize that the ripple width influences ripple pattern discrimination due to the increased ripple depth in the excitation pattern, which occurs as a result of more prominent inter-ripple gaps appearing in spectra with narrow ripples.

**Fig 5:**
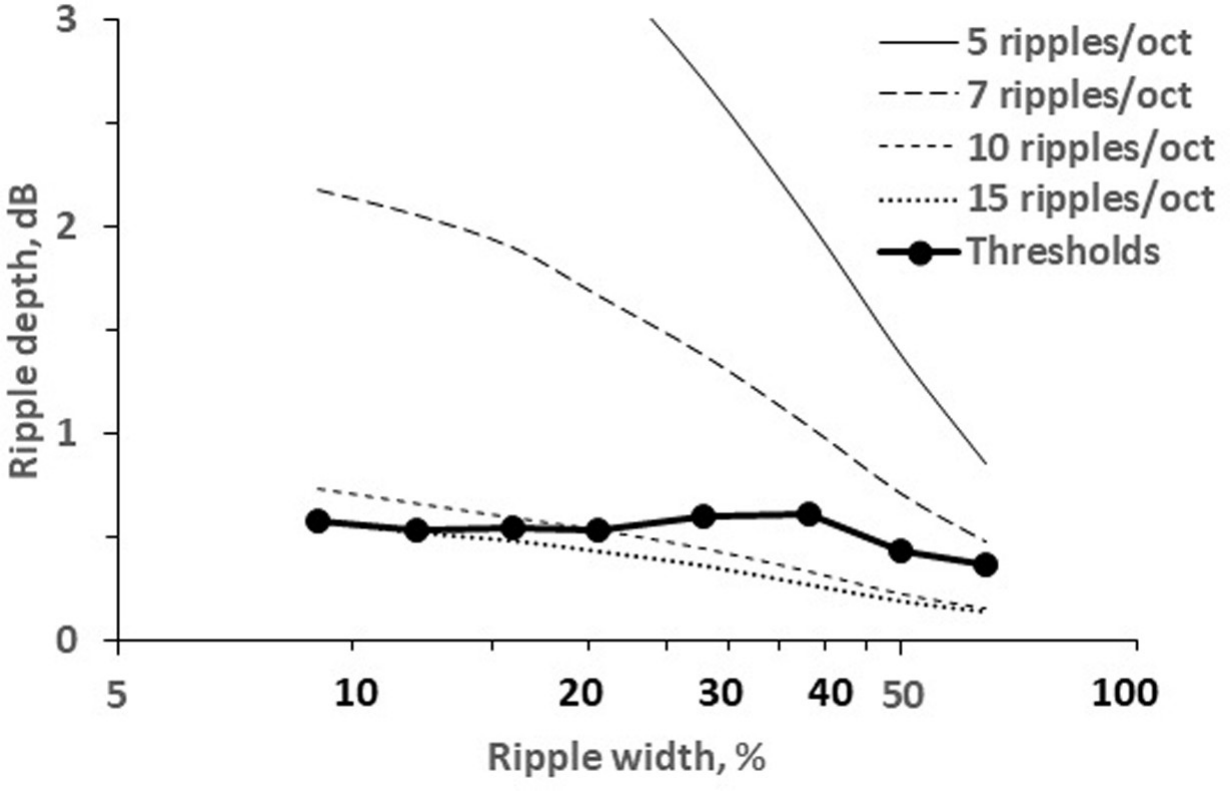
Ripple depth as a function of ripple width in the simulated excitation patterns. The ripple density (ripples/oct) is indicated in the legend. *Thresholds*–ripple depths for experimentally determined thresholds.

Fig 5 shows the depths in the simulated excitation patterns for several ripple densities, ranging from 5 to 15 ripples/oct, as a function of the ripple width. The results of computation demonstrated that the larger the ripple width and the higher the ripple density are, the lower the simulated ripple depth is. These functions allowed to find threshold ripple depth for various combinations of stimulus parameters.

Using the same computation, ripple depths in the excitation patterns were found for threshold stimuli, similar to previous investigations (e.g., [20]). For ripple widths of 9 to 38%, the ripple depths in the simulated excitation patterns ranged from 0.53 to 0.61 dB, with a mean and standard deviation of 0.57 ± 0.04 dB. At ripple widths of 50 and 64%, the simulated ripple depths were a little lower: 0.43 and 0.36 dB, respectively. Thus, in agreement with the general idea of the model, in the excitation pattern the dependence of the ripple depth on the ripple width was negligible for the most part of the width range.

### Temporal cues

Rippled stimuli have a hidden temporal organization, with an increased probability of repeated waveforms after delays of 1/*δf*, where *δf* is the ripple spacing in linear frequency units. This temporal organization manifests in the autocorrelation function (ACF) of a rippled stimulus. In addition to a non-delayed (zero lag) segment, the ACF has a delayed segment with a lag of 1/*δf*, whereas the ACF of a non-rippled stimulus has no delayed segment. This rippled noise structure could be a cue for the resolution of rippled spectrum patterns based on the temporal mechanism of frequency analysis. Temporal analysis of rippled stimuli has been suggested in a number of studies [1, 4, 5, 21, 22].

**Fig 6:**
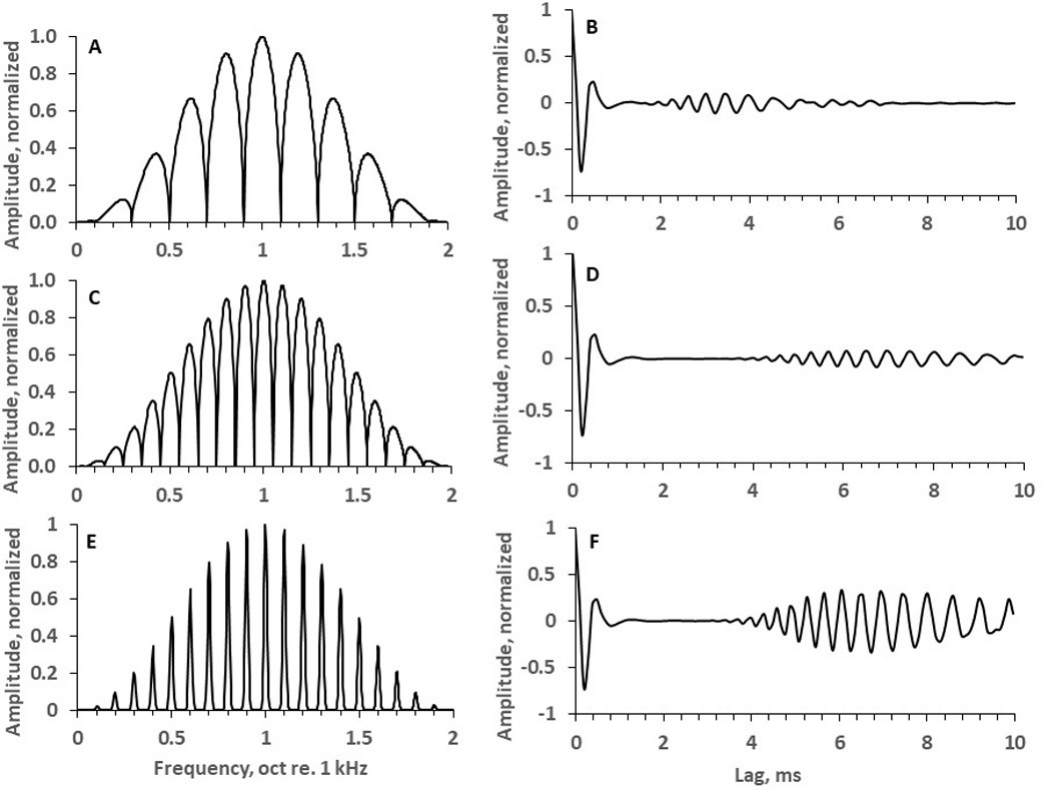
Rippled spectra with various ripple widths and respective ACFs. A: ripple density of 5 ripples/oct and ripple width of 64%. B: ACF of the signal, normalized relative the peak of the non-delayed segment. C and D: the same for 10 ripples/oct and a ripple width of 64%. E and F: the same for 10 ripples/oct and a ripple width of 16%.

Fig 6 shows ACFs of signals with resolvable and unresolvable rippled spectrum patterns. For a frequency band of 1 to 4 kHz (the bandwidth used in the present study) and a ripple density of 5 ripples/oct (Fig 6A), the ripple density expressed in ripples/kHz varied from 7 ripples/kHz at the 1-kHz spectrum edge to 1.75 ripples/kHz at the 4-kHz spectrum edge; thus, the delayed ACF segment occurred from 1.75 to 7 ms (Fig 6B). This ripple density allowed for discrimination between the rippled test signal and the non-rippled reference signal at all the tested ripple widths, ranging up to 64% (see Fig 3). At a ripple density of 10 ripples/oct (Fig 6C), the lag of the delayed segment was twice as long as it was at a ripple density of 5 ripples/oct; thus, this segment appeared from 3.5 to 14 ms (Fig 6D). At a ripple width of 64% and a ripple density of 10 ripples/oct, a rippled test signal and a non-rippled reference signal could not be distinguished (see Fig 3). These discrimination abilities showed that the delayed segment of the ACF in Fig 6B was within the limit where the temporal organization could be recognized, whereas the delayed segment of the ACF in Fig 6D was beyond this limit (the exact value of this limit could not be specified because it was not known which part of the delayed ACF segment was sufficient for detecting temporal organization). For narrow ripples (Fig 6E), a ripple pattern with a density of 10 ripples/oct was resolvable (see Fig 3); in this case, the ratio of the delayed-to-non-delayed ACF segments was higher than that for wider ripples (Fig 6F). This dependence of the resolution on the ripple width is possible if the increase in the ratio of the delayed-to-non-delayed ACF segments (i.e., the ratio of the autocorrelated to uncorrelated components of the input signal) due to narrower ripples results in a longer limit for recognizing the temporal structure.

In previous studies, the ACF lag limits were found to depend on the frequency of the signal, ranging from 25 ms for a center frequency of 0.5 kHz to 12 ms for a center frequency of 4 kHz [14]. The present study extends these estimates, showing that the limit also depends on the ratio of the structured-to-random components of the rippled signal. Assuming, as a first approximation, that this limit is equal to the ripple density at the center frequency of the signal, this limit varies from 68 ms for ripple widths of 20% or shorter to 6.5 ms for a ripple width of 64%. It is possible that the range is even wider for different ripple forms. In this case, the range of the limit variation could include the range of the fundamental frequency discrimination, from 25 ms for a frequency of 0.5 kHz to 5 ms for a frequency of 4 kHz [23].

## Conclusion

Ripple density resolution based on spectrum processing is influenced by the ripple width because of variations in the inter-ripple gaps: the narrower the ripples are, the wider the gaps, and the higher the resolution. Ripple density resolution based on temporal processing is influenced by the ripple width because of variations in the ratio of the correlated-to-uncorrelated components: the narrower the ripples are, the larger the ratio, and the higher the resolution.
